# A dual-scale micro/nanofibrous PLGA scaffold fabricated by co-electrospinning for regenerative endodontic procedures

**DOI:** 10.1590/1678-7765-2026-0524

**Published:** 2026-09-11

**Authors:** Stephanie Isabel Diaz Zamalloa, Caroline Carvalho dos Santos, Leticia Martins Santos, Victor Elias Arana-Chavez, Fernando Neves Nogueira, Carla Renata Sipert, Celso Luiz Caldeira

**Affiliations:** 1 Universidade de São Paulo Faculdade de Odontologia Departamento de Endodontia São Paulo Brasil Universidade de São Paulo, Faculdade de Odontologia, Departamento de Endodontia, São Paulo, Brasil; 2 Universidade de São Paulo Faculdade de Odontologia Departamento de Biomateriais e Biologia Oral São Paulo Brasil Universidade de São Paulo, Faculdade de Odontologia, Departamento de Biomateriais e Biologia Oral, São Paulo, Brasil

**Keywords:** Polymers, Regenerative endodontics, Scaffolds, Stem cells

## Abstract

**Introduction:**

The unpredictability of blood clots as natural scaffolds in regenerative endodontic procedures (REPs) has driven the search for synthetic alternatives with reproducible properties. This study aimed to develop and characterize a dual-scale micro/nanofibrous poly(lactic-co-glycolic acid) (PLGA) scaffold fabricated by co-electrospinning and evaluate its physicochemical and biological suitability for REPs under lipopolysaccharide (LPS)-induced inflammatory conditions.

**Methodology:**

A 7.5% (w/v) PLGA solution was co-electrospun using distinct parameters (flow rate and needle-to-collector distance) to generate a dual-scale fiber architecture. Physicochemical characterization included water uptake, *in vitro* degradation, and morphological analysis by scanning electron microscopy (SEM). Biological properties were evaluated using human apical papilla cells (APCs). Metabolic activity (Alamar Blue), mineralization (Alizarin Red S), and cell adhesion/morphology (SEM) were assessed in the presence or absence of LPS to simulate an inflammatory environment.

**Results:**

The co-electrospun scaffold showed a hierarchical structure with interconnected micro and nanofibers. Water uptake reached 147.4% within 24 h and increased gradually thereafter. The scaffold showed a biphasic degradation profile—slow initial degradation (21.5% over 28 days) followed by accelerated loss (63.1% at day 45). Hydration induced fiber swelling and pore remodeling. The scaffold supported APC adhesion, spreading, and metabolic activity over 72 h. Under LPS stimulation, APCs maintained metabolic activity and showed robust mineralization potential after 21 days, comparable to the positive control.

**Conclusion:**

The co-electrospun PLGA scaffold showed physicochemical properties compatible with tissue ingrowth and supported APCs function under inflammatory conditions. By providing a controlled microenvironment, it represents a potential scaffold design for future REPs.

## Introduction

Regenerative endodontic procedures (REPs) have shifted the management of immature permanent teeth with necrotic pulp toward restoration of a functional pulp–dentin complex.^1^ Current protocols rely on the induced blood clot as a scaffold. However, its formation is highly variable and often lacks the structural stability, controlled architecture, and cell density required for predictable tissue regeneration.^2,3^

Alternative biologically derived scaffolds, including platelet concentrates such as platelet-rich plasma and platelet-rich fibrin, have shown the ability to enhance cell recruitment and release bioactive molecules. Nevertheless, their clinical use remains associated with limitations (including donor-dependent variability, relatively fast biodegradation kinetics, limited control over structural properties, poor sensitivity handling, and reduced capacity to maintain a stable three-dimensional architecture over time).^4,5^ These limitations have encouraged the development of engineered scaffolds with improved reproducibility and physicochemical control.

Synthetic biomaterials have emerged as promising alternatives because they enable controlled modulation of architecture, degradation kinetics, porosity, and mechanical properties.^6,7^ Among them, electrospinning can fabricate fibrous scaffolds that mimic the extracellular matrix, promoting cell adhesion, migration, and differentiation by biomimetic topographical cues.^8-13^ Co-electrospinning, in particular, permits the development of multi-scale architectures with tailored fiber arrangements that more closely reproduce native tissue complexity and improve structural stability and surface interactions.^14-18^ Poly(lactic-co-glycolic acid) (PLGA) is a widely investigated synthetic polymer with adjustable degradation rates, favorable mechanical properties, and established biocompatibility, making it particularly attractive for regenerative endodontic applications.^12,19^ However, despite these advantages, conventional electrospun PLGA scaffolds may still face limitations related to fiber organization, degradation behavior, and biological performance under inflammatory conditions, reinforcing the need for scaffold optimization strategies.

Because REPs are commonly performed in teeth with previous infection and persistent inflammatory stimuli, scaffold performance should be evaluated under conditions that better reproduce the clinical microenvironment. Lipopolysaccharide (LPS), a major component of Gram-negative bacterial cell walls frequently associated with endodontic infections, can modulate stem cell behavior by affecting proliferation, migration, differentiation, and inflammatory signaling pathways.^20^ Human apical papilla cells (APCs), which play a central role in root development and regenerative outcomes, may, therefore, respond differently under inflammatory conditions, making this model particularly relevant for translational investigation.^21-23^

Accordingly, this study aimed to develop and characterize a co-electrospun PLGA scaffold with dual-scale fiber architecture and to assess its physicochemical and biological performance using APCs, including under inflammatory conditions induced by LPS.

## Methodology

This study was approved by the Ethics Committee of the School of Dentistry, University of São Paulo (CAEE: 79390524.4.0000.0075; Protocol #6.808.699).

### Scaffold Fabrication

PLGA (50:50; Mw 76,000–115,000 g/mol; Purasorb 5010, Purac Biomaterials, The Netherlands) was dissolved at 7.5% (w/v) in hexafluoro-2-propanol (HFIP - Sigma-Aldrich, UK) under overnight stirring. The solution was loaded into two 10-mL syringes fitted with 22-gauge needles and simultaneously co-electrospun using a custom electrospinning system (3DBS, Brazil). The two syringes operated concurrently under distinct processing parameters to generate an integrated dual-scale fiber architecture composed of micro- (18-cm working distance; 1.0-mL/h flow rate) and nanofibers (12-cm working distance; 0.8-mL/h flow rate) that were simultaneously deposited onto the same rotating collector. A 11.8-kV voltage and a 350-rpm collector speed were applied under controlled conditions (22°C, 57% humidity).

**Figure 1** provides a schematic overview of the fabrication process.

**Figure 1:**
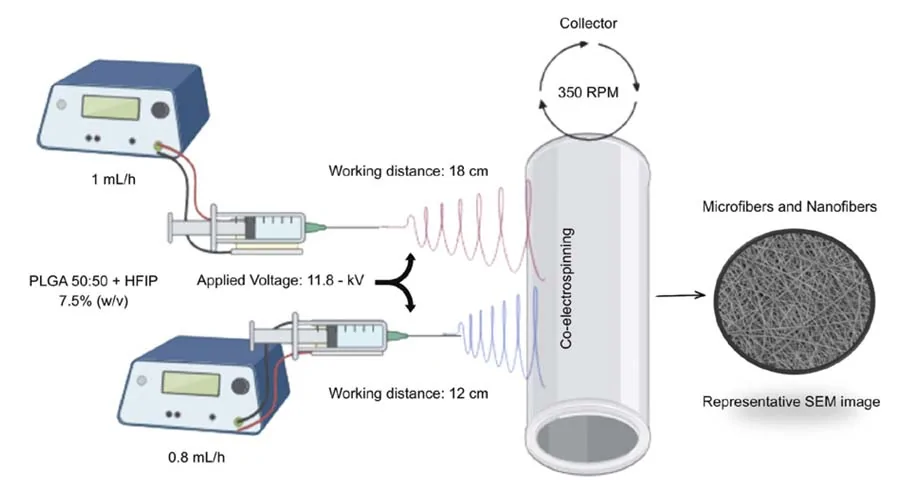
Schematic representation of the fabrication of the co-electrospun 7.5% PLGA scaffold illustrating the simultaneous deposition of micro- and nanofibers using distinct electrospinning parameters to generate an integrated dual-scale architecture.

Membranes were vacuum-dried overnight, stored at 4°C, and punched into 10-mm diameter discs for all assays. Biological and physicochemical assays were generally performed in triplicates unless otherwise stated.

Before the comprehensive physicochemical and biological characterization, three PLGA scaffold architectures were preliminarily evaluated during scaffold development: an integrated dual-scale scaffold fabricated by co-electrospinning, a microfibrous scaffold, and a bilayer dual-scale scaffold obtained by assembling independently fabricated micro- and nanofibrous membranes using gelatin as an adhesive layer. Human APCs were seeded onto each scaffold architecture, while APCs cultured directly on the well surface without a scaffold served as the control group. Metabolic activity was evaluated after 24 h using the AlamarBlue assay. Absorbance was measured at 560 and 600 nm (Synergy H1, Biotek, Winooski, VT, USA), and AlamarBlue reduction was calculated and normalized to the control group. This preliminary evaluation was performed to support the selection of the scaffold for the subsequent physicochemical and biological characterization.

### Physicochemical Characterization

#### Swelling Behavior

The swelling behavior of the scaffolds was evaluated gravimetrically under physiologically relevant conditions. Dry discs (Wd) were immersed in 1 mL phosphate-buffered saline (PBS; pH 7.4) at 37°C and weighed (Ws) at predefined time points (1, 5, and 10 min; 2 and 24 h; 7, 15, 28, and 45 days). Before weighing, samples were gently removed from the solution, and excess surface liquid was carefully removed using absorbent paper to minimize the influence of non-adherent superficial fluid. Swelling (%) was calculated as: (Ws−Wd)/Wd×100 .^24^

#### In Vitro Degradation

*In vitro* degradation was assessed by mass loss. The initial dry weight of discs (Wo) was recorded, and samples were incubated in 1 mL PBS (pH 7.4) at 37°C under static conditions. The degradation medium was replaced every 48 h using freshly prepared PBS adjusted to pH 7.4 to maintain buffered conditions and minimize the accumulation of acidic degradation byproducts. At predetermined time points (7, 15, 28, and 45 days), samples were vacuum-dried overnight and weighed (Wd). Mass loss (%) was calculated as: ( Wo −Wd)/ Wo ×100 .^25^

#### Morphological Analysis

Scaffold morphology was evaluated using scanning electron microscopy (SEM; LEO 440i, Cambridge, UK). Samples were mounted on aluminum stubs, sputter-coated with gold, and imaged at multiple magnifications (×100, ×500, ×750, ×1500, ×2000, and ×5000). Fiber diameter and pore size were quantified from SEM micrographs acquired at ×5000 magnification on ImageJ (National Institutes of Health, Bethesda, MD, USA). Measurements were performed using three randomly selected images per sample. For each image, 50 measurements of fiber diameter and 50 measurements of pore size were obtained from randomly selected regions. Because the scaffold showed an integrated dual-scale architecture with intermingled micro- and nanofibers, measurements were performed collectively rather than by fiber population. Samples subjected to degradation for 45 days were prepared and evaluated using the same protocol to assess morphological changes over time.

## Biological Characterization

### Preparation of Scaffold Holders (PLA Rings)

To secure the membranes at the bottom of culture wells and prevent floating, custom-made polylactic acid (PLA) rings were fabricated using fused deposition modeling 3D printing (Meshmixer and Repetier Host software). Rings were designed with a 10-mm external diameter, a 6-mm internal diameter, and a 2-mm thickness. Printing was performed with a bed temperature of 60°C and an extruder temperature of 190°C.

### Sterilization and Pre-conditioning

PLGA membranes and PLA rings were sterilized by immersion in 70% ethanol, followed by exposure to ultraviolet light for 30 min per side. Subsequently, membranes were pre-wetted in PBS for 24 h at 37°C to enhance cell penetration and mimic physiological conditions prior to cell seeding.

### APC Culture and Characterization

APCs were obtained from extracted third molars (with informed consent) using an explant culture technique. Briefly, the apical papilla was carefully dissected, minced into small fragments (approximately 1–2 mm^3^), and placed into 25-cm^2^ culture flasks with a minimal volume of proliferation medium (PM) to facilitate tissue attachment. PM consisted of an alpha-minimum essential medium (α-MEM; Sigma-Aldrich, St. Louis, MO, USA) supplemented with 10% fetal bovine serum (FBS; Gibco), 100 U/mL penicillin, 100 μg/mL streptomycin, and 0.5 μg/mL amphotericin B (Invitrogen). Cultures were maintained at 37°C in a humidified atmosphere of 5% CO₂ and 95% air. Upon reaching 80% confluence, cells were subcultured. All experiments were performed using cells from passages 4 to 6.^26,27^

To confirm mineralization potential, APCs were seeded in 24-well plates at a 5 × 10^4^-cells/well density. After reaching confluence, cells were cultured for 21 days in PM (negative control) or a differentiation medium (DM). DM consisted of PM supplemented with 2 mM KH₂PO₄ and 100 nM dexamethasone. Media were refreshed every two days. Mineralization was assessed by Alizarin Red S staining. Cells were fixed with 4% paraformaldehyde, stained with 40 mM Alizarin Red S solution (pH 4.2; Sigma-Aldrich) for 30 min, and rinsed. For quantification, the stain was eluted with 500 μL of 10% ammonium hydroxide (Êxodo, Sumaré, Brazil) per well, and absorbance was measured at 405 nm (Synergy H1, Biotek, Winooski, VT, USA).

### Cell Metabolic Activity

APCs were seeded onto the PLGA membranes (secured by PLA rings) in 48-well plates at a 1.5 × 10^4^-cells/well density with or without 1 μg/mL lipopolysaccharide (LPS; from *E. coli* O111:B4, L4391, Sigma-Aldrich).^28,29^Cells were cultured in 500 μL of PM for 24, 48, and 72 h. At each time point, metabolic activity was assessed by adding 40 μL of AlamarBlue reagent (10% v/v in PM; Invitrogen) to each well. After 4 h of incubation under standard conditions (protected from light), 100 μL of the supernatant was transferred to a black 96-well plate. Fluorescence was measured at 530-nm excitation and 590-nm emission (Synergy H1, Biotek, Winooski, VT, USA). Background fluorescence from the scaffold material was subtracted using cell-free controls (membrane incubated with AlamarBlue under identical conditions).

### Mineralization Potential

APCs were seeded onto membranes (secured by PLA rings) in 48-well plates at a 1.5 × 10^4^-cells/well density with or without 1 μg/mL LPS. Cells were cultured for 21 days in either PM or DM, with media refreshed every two days. At the endpoint, cells were fixed with 4% paraformaldehyde for 30 min, rinsed with PBS, and stained with 40 mM Alizarin Red S solution (pH 4.2) for 30 min. After macroscopic imaging, the stain was eluted with 200 μL of 10% ammonium hydroxide per well. Absorbance was measured at 405 nm (Synergy H1, Biotek, Winooski, VT, USA). Values were normalized to the positive control (DM without LPS). Background staining from acellular scaffolds was subtracted.

### Cell Adhesion and Morphology

APCs were seeded onto membranes (secured by PLA rings) in 48-well plates at a 1.5 × 10^4^-cells/well density and cultured in PM for 24 h. Samples were fixed with 2.5% glutaraldehyde in 0.1 M cacodylate buffer (pH 7.2) for 2 h at 4°C, then washed with 0.1 M cacodylate buffer. Dehydration was performed by a graded ethanol series (30, 50, 70, 80, 90, and 100%), followed by chemical drying with hexamethyldisilazane (HMDS; Electron Microscopy Science, Hatfield, PA, USA) for 10 min. Specimens were mounted on stubs, sputter-coated with gold, and examined under SEM (LEO 440i) at ×100, ×250, and ×750 magnifications. Cell morphology and adhesion were qualitatively assessed, and cell length was measured on ImageJ on all well-defined cells within the micrographs.

## Data Analysis

Data are shown as mean ± standard deviation (SD) from at least three independent experiments performed in triplicates. Analyses were conducted on GraphPad Prism 9.0 (GraphPad Software, San Diego, CA, USA). Normality was assessed by the Shapiro–Wilk test. For comparisons between two groups, the unpaired Student *t*-test was applied. For comparisons involving three or more groups, one-way or two-way analysis of variance (ANOVA) was used, followed by Tukey’s post hoc test for multiple comparisons. When normality was not met, the Mann–Whitney U test was used. Correlation analyses were performed using Spearman’s rank correlation coefficient. Statistical significance was set at *P* < 0.05.

## Results

### Preliminary Evaluation of PLGA Scaffold Architectures

Overall, three PLGA scaffold architectures were preliminarily evaluated during scaffold development to support scaffold selection for this study, with APCs cultured without a scaffold serving as the control group. The integrated dual-scale scaffold fabricated by co-electrospinning showed significantly greater metabolic activity than the microfibrous scaffold, bilayer dual-scale scaffolds, and control group after 24 h of culture (P < 0.05) (**Figure 2**). Based on these preliminary biological findings, its integrated architecture, and simpler single-step fabrication, the co-electrospun dual-scale scaffold was selected for the subsequent physicochemical and biological characterization.

**Figure 2:**
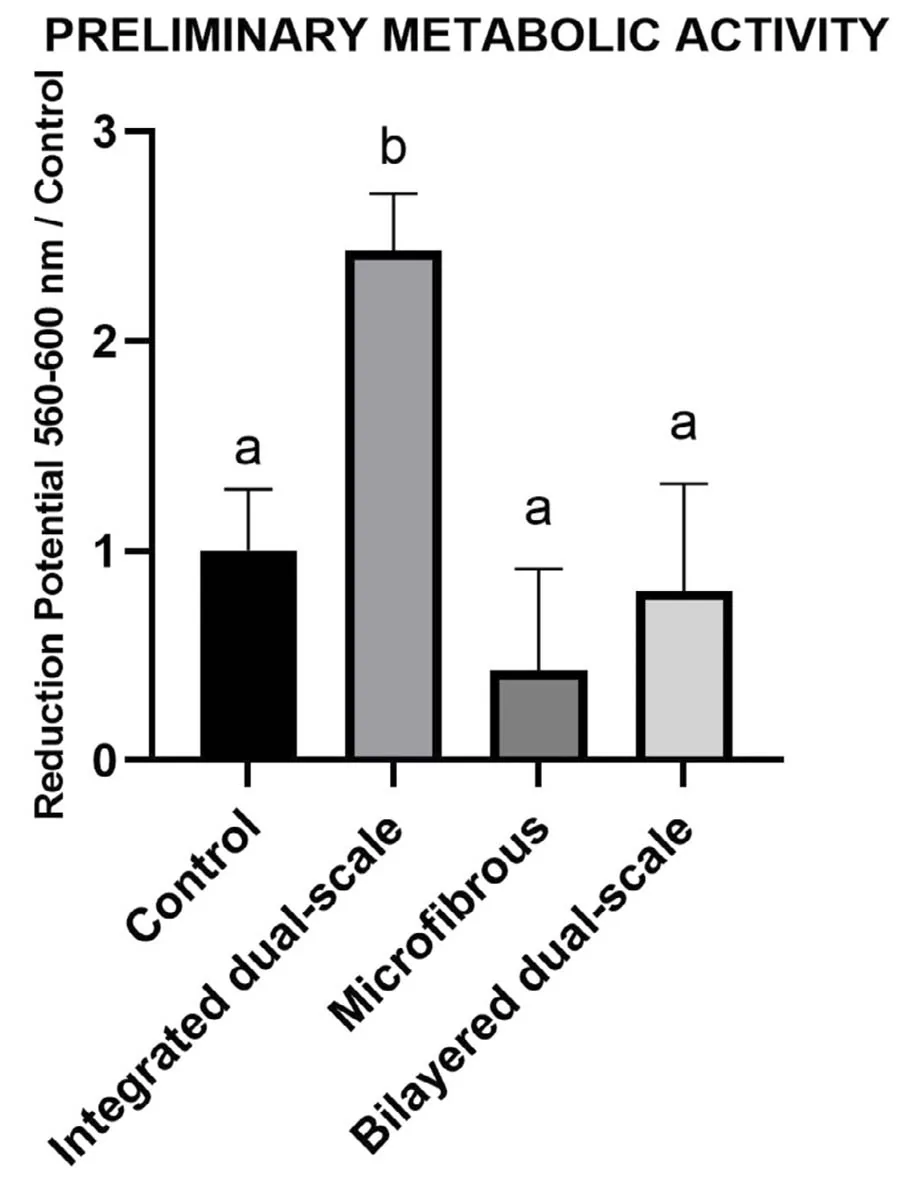
Preliminary metabolic activity of three PLGA scaffold architectures evaluated during scaffold development. Human apical papilla cells (APCs) were cultured for 24 h on an integrated dual-scale scaffold fabricated by co-electrospinning, a microfibrous scaffold, and a bilayer dual-scale scaffold assembled from independently fabricated micro- and nanofibrous membranes using gelatin as an adhesive layer. APCs cultured directly on the well surface without a scaffold served as the control group. Metabolic activity was evaluated using the AlamarBlue assay. Different lowercase letters indicate statistically significant differences between groups (one-way ANOVA followed by Tukey’s post hoc test, *P* < 0.05).

### Physicochemical characterization

#### Swelling Behavior and Macroscopic Changes

The co-electrospun PLGA scaffold showed rapid initial swelling followed by progressive hydration over longer incubation periods. During the short-term evaluation, scaffold swelling increased markedly within the first minutes, reaching 90.2% after 10 min and continuing to rise to approximately 147.4% after 24 h (one-way ANOVA, *P*<0.05) (**Figure 3A**). In the independent long-term evaluation (up to 45 days), swelling values ranged from 18.3% at day 7 to 179.2% at day 45. However, no statistically significant differences were observed between time points (one-way ANOVA, *P* > 0.05) (**Figure 3B**). Macroscopically, hydrated scaffolds showed increased flexibility and progressive structural deformation over time, becoming more pliable during incubation (**Figure 3C**).

**Figure 3:**
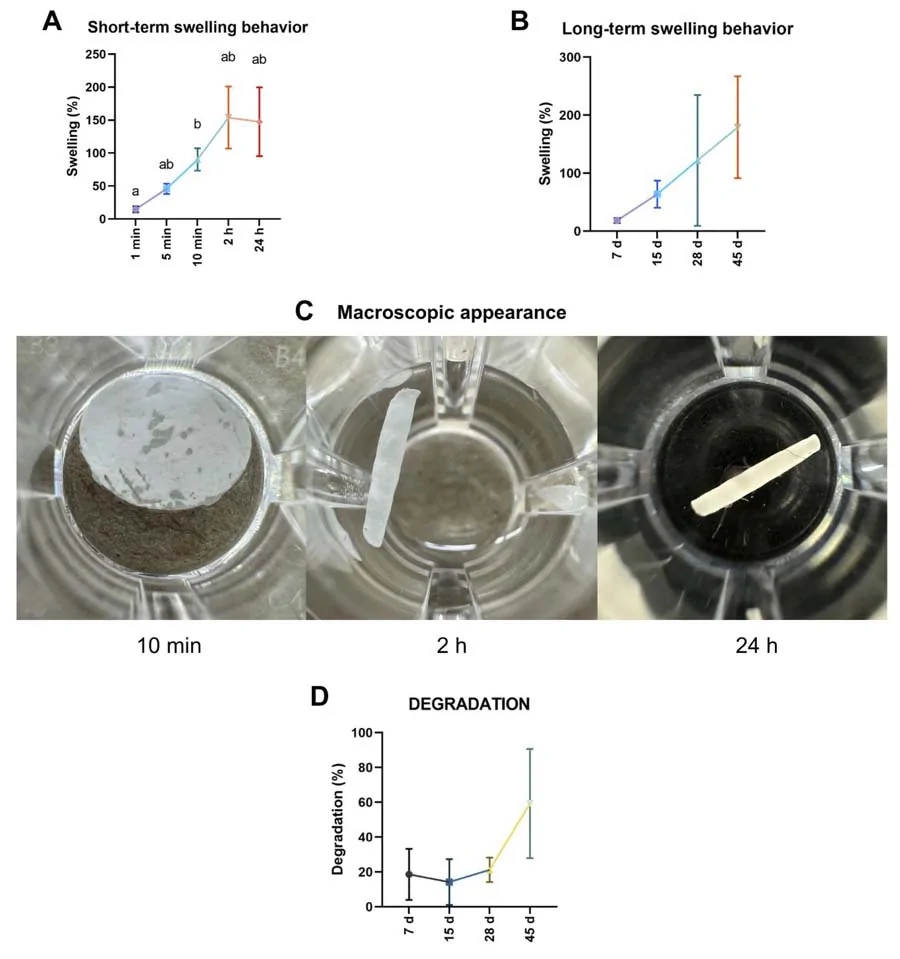
Swelling behavior, degradation profile, and macroscopic changes of the co-electrospun 7.5% PLGA scaffold during incubation in PBS. (A) Short-term swelling behavior from 1 min to 24 h. Different lowercase letters indicate statistically significant differences between time points (one-way ANOVA, *P* < 0.05). (B) Long-term swelling behavior evaluated up to 45 days. (C) Macroscopic appearance of the scaffold after 10 min, 2 h, and 24 h of incubation in PBS. (D) *In vitro* degradation profile assessed by gravimetric mass loss over 45 days.

#### In Vitro Degradation

The scaffold showed relatively limited mass loss during the first 28 days of incubation, remaining below 21.5% throughout this period. Greater mass loss was observed from days 28 to 45, reaching 63.1% at the final point (**Figure 3D**). Despite this trend toward increased degradation over time, no statistically significant differences were detected between time points (one-way ANOVA, *P* > 0.05).

#### Morphological Characterization

SEM analysis found a randomly oriented and interconnected fibrous network with an integrated dual-scale architecture at baseline. Initially, scaffolds showed a 0.65-µm fiber diameter (0.33–1.25 µm) and a 4.72-µm pore size (0.71–9.03 µm) (**Figure 4A–C**).

**Figure 4:**
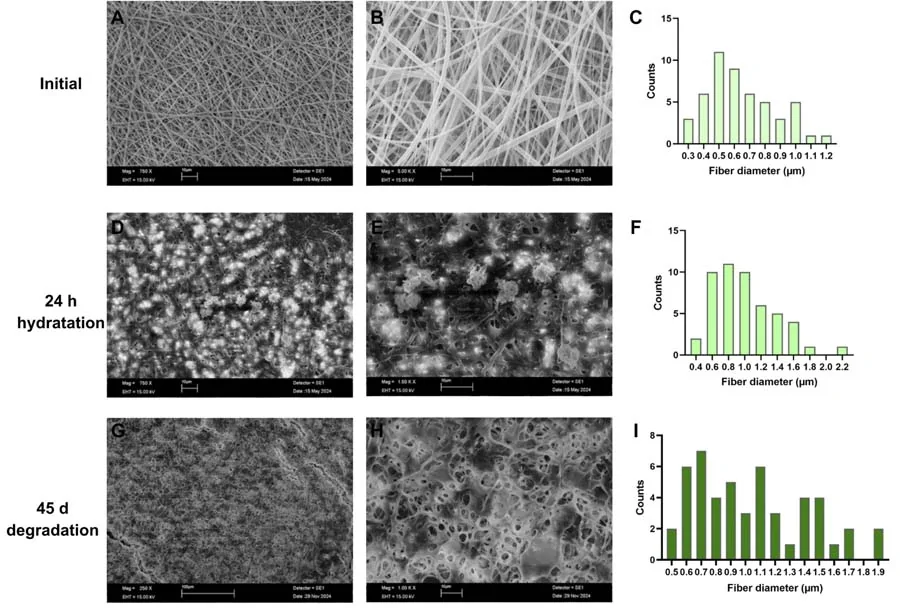
Representative SEM micrographs showing the structural evolution of the co-electrospun dual-scale PLGA scaffold during hydration and degradation in PBS. Initial morphology: (A) low-magnification image (×750), (B) high-magnification image (×5000), and (C) fiber diameter distribution. After 24 h of hydration: (D) low-magnification image (×750), (E) intermediate-magnification image (×1500), and (F) fiber diameter distribution. After 45 days of degradation: (G) low-magnification image (×250), (H) intermediate-magnification image (×1000), and (I) fiber diameter distribution.

After 24 h of incubation, SEM images showed hydration-associated morphological changes characterized by increased apparent fiber diameter, closer fiber packing, increased tortuosity, and reduced pore size, whereas the fibrous architecture remained identifiable (**Figure 4D–F**). Quantitatively, fiber diameter increased to 1.02 µm (0.46–2.16 µm), whereas pore size decreased to 1.91 µm (0.48–6.76 µm).

After 45 days, more pronounced structural alterations became evident, including fiber fusion and partial loss of the original fibrous organization, suggesting progressive remodeling of the scaffold architecture over time (**Figure 4G–I**). At this stage, fiber diameter reached 1.08 µm (0.46–3.63 µm), whereas pore size increased to 3.50 µm (0.92–8.37 µm).

Quantitative comparison between baseline and 45-day samples showed significantly larger fiber diameters after degradation (Mann–Whitney test, *P* < 0.05) (**Figure 5A**), whereas pore size significantly decreased when compared with baseline values (Mann–Whitney test, *P* < 0.05) (**Figure 5B**).

**Figure 5:**
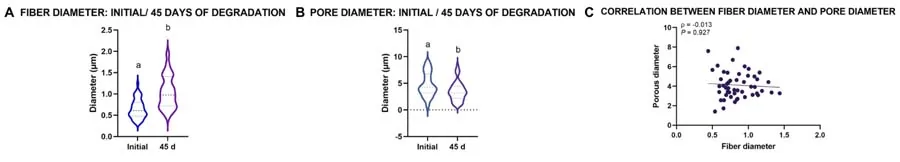
Quantitative structural analysis of the co-electrospun dual-scale PLGA scaffold before and after degradation. (A) Fiber diameter distribution comparing initial scaffolds and after 45 days of degradation. (B) Pore size distribution comparing initial scaffolds and after 45 days of degradation. Different lowercase letters indicate statistically significant differences between groups (Mann–Whitney test, *P* < 0.05). (C) Correlation between fiber diameter and pore size.

No significant correlation was observed between fiber diameter and pore size (Spearman’s ρ = −0.013, P = 0.927), suggesting independent variation of these structural parameters during scaffold remodeling (**Figure 5C**).

## Biological Characterization with APCs

### APCs Functional Characterization

APCs showed osteo/odontogenic potential, with increased Alizarin Red S staining and greater calcium deposition after 21 days in DM than in PM (unpaired t test, P < 0.05) (**Figure 6A,B**).

**Figure 6:**
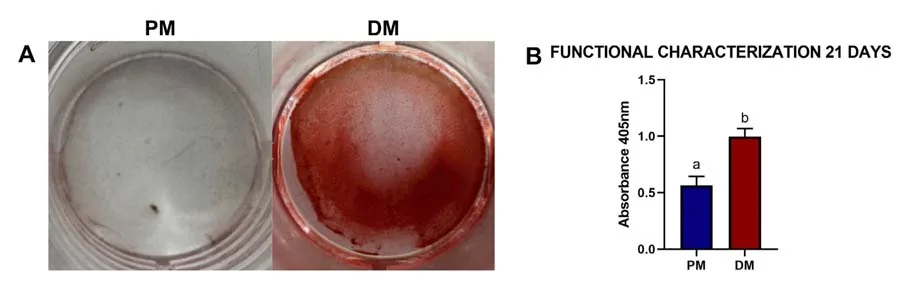
Functional characterization of apical papilla cells (APCs). (A) Representative Alizarin Red S staining of APCs cultured in proliferation (PM) and differentiation media (DM) for 21 days. (B) Quantification of mineralized matrix deposition after 21 days, expressed as absorbance at 405 nm. Different lowercase letters indicate statistically significant differences between groups (*P* < 0.05).

### Metabolic Activity Under Inflammatory Challenge

The metabolic activity of APCs cultured on the PLGA scaffold was assessed over 72 h using the AlamarBlue assay. At all time points, cells seeded on the scaffold showed fluorescence levels comparable to the control group in the presence and absence of LPS stimulation (P > 0.05) (**Figure 7A**).

**Figure 7:**
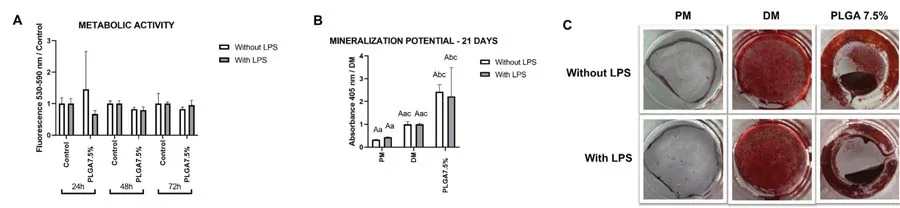
Biological performance of the co-electrospun 7.5% PLGA scaffold under inflammatory conditions. (A) Metabolic activity of APCs cultured under control conditions or in contact with the scaffold, evaluated by Alamar Blue assay at 24, 48, and 72 h in the presence or absence of LPS. (B) Quantitative analysis of mineralized matrix deposition after 21 days under culture conditions [proliferation (PM) and differentiation media (DM) and scaffold] with or without LPS stimulation. Uppercase letters indicate statistically significant differences between LPS conditions within the same experimental group, whereas lowercase letters, statistically significant differences between culture conditions (PM, DM, and scaffold) (*P* < 0.05). (C) Representative Alizarin Red S staining illustrating mineral deposition under culture conditions according to LPS exposure.

### Mineralization Potential Under Inflammatory Challenge

After 21 days, APCs seeded on the PLGA scaffold in DM showed substantial mineral deposition, as evidenced by Alizarin Red S staining and quantification. Mineralization levels in the scaffold groups with or without LPS were comparable to those of the positive control, with no statistically significant differences (two-way ANOVA, P > 0.05). Cells cultured in PM showed minimal staining (**Figure 7B,C**).

### APCs Adhesion and Morphology

SEM analysis after 24 h found that APCs adhered and colonized the PLGA scaffold surface. Cells showed a heterogeneous morphology, with lengths ranging from 20 to 120 µm (median ~60 µm) (**Figure 8A**), forming clustered, elongated, spindle-shaped, island-like structures (**Figure 8B-E**). Additionally, cells extended thin cytoplasmic projections, consistent with filopodia, which seem to anchor them to the underlying nanofibers (**Figure 8E,** yellow arrows).

**Figure 8:**
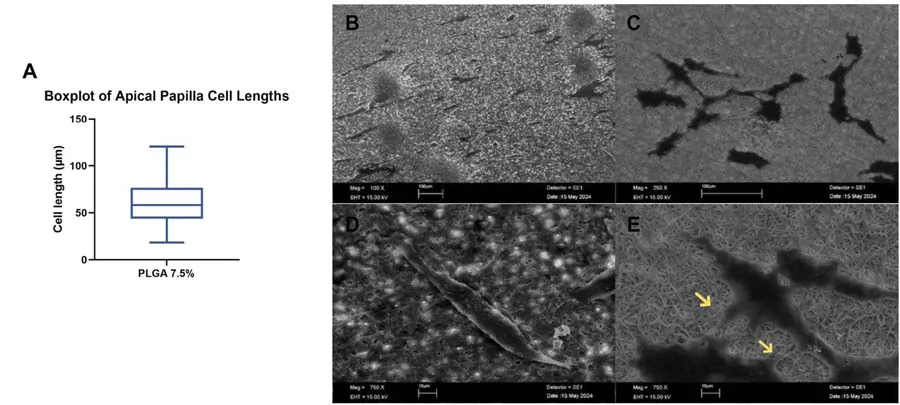
Adhesion and morphological characterization of APCs cultured on the co-electrospun 7.5% PLGA scaffold. (A) Quantitative analysis of APC length distribution after culture on the scaffold. (B–E) Representative SEM micrographs showing cell adhesion, spreading, elongated morphology, and cytoplasmic extensions interacting with the fibrous architecture. Arrows indicate filopodia-like cellular projections and cell–scaffold interactions.

## Discussion

The development of scaffolds for REPs remains critical, particularly given the clinical challenges associated with achieving predictable apical bleeding and maintaining a stable scaffold within the root canal environment.^30^ This study developed a co-electrospun PLGA scaffold with an integrated dual-scale fiber architecture and evaluated its physicochemical and biological performance using human APCs under LPS-induced inflammatory conditions. Overall, the scaffold showed characteristics that support its potential application in REPs.

The scaffold showed progressive degradation behavior: relatively limited mass loss during the first 28 days followed by increased mass loss at later time points, reaching 63.1% at day 45. Although no statistically significant differences were detected between time points, these findings suggest maintenance of scaffold integrity during the early stages followed by gradual structural remodeling over time. Since PLGA degradation is influenced by hydrolysis, polymer composition, and scaffold architecture, the observed behavior is likely associated with the physicochemical characteristics of the material and fabrication process.^31-33^ Additionally, rapid scaffold hydration (147.4% within 24 h) and increased pliability may favor adaptation to the hydrated root canal environment and facilitate interaction with surrounding tissues.

The scaffold in this study was selected following a preliminary evaluation of three PLGA scaffold architectures. The integrated dual-scale scaffold fabricated by co-electrospinning showed greater metabolic activity during the preliminary biological evaluation and provided a simpler single-step fabrication process than the bilayer dual-scale construct assembled after independent electrospinning and gelatin-mediated assembly. These preliminary findings supported its selection for the subsequent physicochemical and biological characterization.

The selected scaffold showed an integrated dual-scale fibrous architecture composed of intermingled co-electrospun nano- and microfibers. This hierarchical organization was designed to resemble the multiscale organization of the native extracellular matrix, which includes structures ranging from nanoscale collagen fibrils to microscale fiber bundles.^8-13^ After hydration, morphological changes (including increased apparent fiber diameter, reduced pore size, and increased fiber tortuosity) were observed, likely reflecting hydration-associated structural rearrangement rather than early scaffold degradation.^34^ After 45 days, more pronounced architectural modifications, including fiber fusion and partial alteration of the original fibrous organization, became evident together with a maintained interconnected fibrous network. Because micro- and nanofibers were intermingled within a single integrated architecture, it is impossible to attribute degradation behavior to individual fiber populations, which should, therefore, be interpreted as representative of overall scaffold remodeling.

The maintenance of an interconnected fibrous organization throughout degradation may be advantageous for regenerative applications since scaffold architecture influences cell attachment, nutrient diffusion, and cellular interactions.^13,15,16,18^ Furthermore, the absence of correlation between fiber diameter and pore size suggests that these structural parameters evolved independently during scaffold remodeling.

Biological evaluation showed excellent compatibility with APCs. Cells adhered and colonized the scaffold surface, showing elongated spindle-shaped morphology with thin cytoplasmic projections consistent with filopodia formation, indicating active spreading and interaction with the scaffold nanotopography.^35,36^ Heterogeneous cell lengths and clustered growth patterns further support favorable cell–scaffold interactions.

Importantly, the APCs cultured on the scaffold maintained metabolic activity comparable to controls at all evaluated time points regardless of LPS stimulation. These findings suggest the absence of cytotoxic effects and the preservation of cellular activity under inflammatory challenges. After 21 days of osteo/odontogenic induction, APCs cultured on the scaffold showed mineralized matrix deposition comparable to positive controls even under LPS, indicating preserved mineralization capacity under inflammatory conditions.

Although Alizarin Red staining showed mineralized matrix deposition, it should not be interpreted as independent evidence of odontogenic differentiation. Future studies including specific odontogenic markers, such as dentin sialophosphoprotein and dentin matrix protein 1, are needed to further characterize the differentiation profile of APCs cultured on the scaffold.

These results corroborate previous reports on the resilience of APCs under inflammatory stimuli and support the ability of the scaffold to sustain cellular function in environments that partially mimic infected root canal conditions.^37-40^ Consistent with previous observations,^22^ the scaffold seems to provide a permissive microenvironment that can support cellular activity even under inflammatory challenges.

Overall, the selected scaffold combines several complementary characteristics, including an integrated dual-scale architecture, a degradation profile compatible with early tissue colonization, preservation of structural continuity during incubation, and maintenance of cellular activity under inflammatory conditions. Together, these characteristics support further investigation of the selected scaffold for REPs.

Despite such encouraging *in vitro* results, several limitations should be acknowledged. First, this study included no mechanical characterization. Although the scaffold showed favorable physicochemical and biological properties, mechanical properties such as tensile strength, elasticity, and structural stability are important for scaffolds intended for REPs. Moreover, the inflammatory model used a single concentration (1 μg/mL) of *E. coli* LPS, selected based on previously validated *in vitro* models of APCs activation.^28,29^ Nevertheless, although this approach provides a standardized and reproducible inflammatory stimulus, it fails to fully reproduce the complexity of infected root canals, which include polymicrobial biofilms, multiple bacterial products, and dynamic host immune responses. Furthermore, degradation analysis was primarily based on gravimetric measurements and selected SEM time points, which are unable to distinguish surface-associated changes, internal degradation mechanisms, or fiber population-specific degradation behavior. Additionally, while SEM findings showed favorable cell attachment and surface colonization, these observations directly assess neither migratory behavior nor cellular infiltration throughout the scaffold architecture. Therefore, the ability of the scaffold to actively promote cellular recruitment and migration remains to be further investigated. Moreover, static culture conditions fail to reproduce dynamic fluid exchanges within the root canal. Future studies should investigate scaffold interactions with multispecies biofilms, inflammatory cells, dynamic culture systems, and migration/infiltration behavior and include comprehensive mechanical characterization to better understand scaffold-guided cellular recruitment during regenerative processes. Ultimately, *in vivo* studies remain necessary to confirm the capacity of the scaffold to support functional pulp–dentin regeneration, angiogenesis, and tissue organization.

## Conclusion

This study shows that a co-electrospun PLGA scaffold with a dual-scale micro/nanofiber architecture showed physicochemical and biological properties in line with REPs demands. The scaffold hierarchical structure was associated with a dynamic microenvironment characterized by rapid hydration, controlled degradation profile conducive to tissue replacement, and the capacity to support APCs adhesion, metabolic activity, and mineralized matrix formation. Importantly, the scaffold sustained these key cellular functions even under inflammatory challenges with LPS, underscoring its potential utility in infected root canals. These findings suggest that this scaffold represents a promising candidate for further investigation in REPs.
